# Effects of face masks on fairness in on-site personnel selection during a pandemic

**DOI:** 10.3389/fpsyg.2023.1168311

**Published:** 2023-08-31

**Authors:** Frank Zinn, Justin Maximilian Mittelstädt

**Affiliations:** Department of Aviation and Space Psychology, Insitute of Aerospace Medicine, Hamburg, Germany

**Keywords:** personnel selection, face mask, cognitive performance, assessment center, communication, COVID-19 pandemic

## Abstract

**Introduction:**

Despite significant challenges, personnel selection procedures had to continue as on-site testing in the Covid-19 pandemic. Health and safety measures and specifically the use of face masks threaten to limit the fairness of cognitive testing and behavioral observation in the assessment center.

**Methods:**

In this study, we compare the performance and pass rates of pilot selection under three different conditions in the selection campaigns of 2019 (pre-pandemic), 2020 (health and safety measures without mask), and 2021 (health and safety measures with mask).

**Results:**

Mask wearing and other health and safety measures had no influence on the objective parameters of pilot selection. However, for some of the areas of competence in the assessment center subjective observability was rated lower for the condition with face masks.

**Discussion:**

We conclude that the fairness and precision of selection processes are not compromised by wearing face masks and that a high degree of standardization in diagnostic instruments prevents a partially reduced subjective observability from affecting the selection’s outcome.

## 1. Introduction

On March 11, 2020, the WHO officially declared the wave of Covid-19 infections, which had previously been considered an epidemic, to be a global pandemic (WHO, 2020). Massive changes in lifestyle became part of daily routines for many months. An economic crisis followed. In the first weeks and months only the most necessary activities were allowed and personal contacts were restricted to the essentials. The Covid-19 pandemic has had a profound effect on many private and professional areas of life. Many areas of business and industry were shut down for weeks or months. Some businesses moved activity to digital platforms where possible. In non-digital private and working life, strict contact rules were imposed. The most visible expressions of public and business Covid-19-related measures were social distancing and face masks worn in public spaces around the world from late April 2020. Like in many other countries, Germany’s federal and state governments decided to tighten mandatory use of masks due to increasing Covid-19 infection rates and the spread of various mutations from January 2021 (Government of the Federal Republic of Germany, Chancellor of the Federal Republic of Germany, 2021). Simple mouth and nose coverings were no longer sufficient; surgical masks or masks conforming to KN95/N95 or FFP2 standards were now obligatory.

In recent years, both cognitive performance tests as well as Assessment Center tasks and interviews have been conducted on digital platforms increasingly, a trend driven by pandemic constraints. But online examinations and online assessments (still) have noteworthy disadvantages. Even if extensive security precautions are implemented, it is not possible to completely exclude the possibility that tests are manipulated (Bloemers et al., 2016; Dendir and Maxwell, 2020).

Apart from the options of manipulation in online tests (Vazquez et al., 2021), home equipment cannot ensure standardized item presentation. Particularly for tests measuring psychomotor skills and hand-foot coordination, precise joysticks and foot pedals are a prerequisite for accurate test application. Calibrated equipment guarantees standardized conditions, another aspect of test fairness (Häusler et al., 2007; Basner et al., 2021).

Especially selection for safety-relevant occupations must exclude the possibility of output falsification. For this purpose, (a final) on-site selection proceeding is currently indispensable.

Moreover, personnel selection traditionally involves face-to-face contact between candidates and potential employers. Typical Assessment Center (AC) exercises primarily test candidates’ social competencies and focus on how the candidate behaves and communicates (Damitz et al., 2003; Hoffman et al., 2015). Many employers seek personal contact with candidates to build trust (Basch et al., 2021) and because they have the impression that they gain a better picture of the candidates (Stone et al., 2015). Candidates likewise perceive on-site interviews as more personal and raise fewer data protection concerns compared to digital online interviews (Langer et al., 2017). Work sample tests are another reason why on-site testing often is reasonable; special equipment or devices are usually required for this purpose, or the work is to be tested in specific environments.

The advantages of on-site examinations and assessments especially for safety-relevant occupations might outweigh the additional constraints and costs generated by meeting the requirements of hygienic measures in a pandemic. Moreover, there might be no alternative for the reasons mentioned above.

Conducting personnel selection during a pandemic that is also compliant with official public health guidelines is a challenge. Administrating standardized computer tests in groups (e.g., cognitive tests) during the pandemic is often only possible by reducing the test capacity in order to maximize the distances between the candidates.

Adopting health and safety measures once again raises questions of fairness. The additional wearing of face masks is perceived by many as uncomfortable. Concerns have been raised about degraded performance and observability – and thus fairness – compared with tests and assessments during non-pandemic times. Norms established in pre-pandemic times might not be applicable to performance data during the pandemic (Gibson et al., 2021).

A survey on surgeons suggest that they are limited in their performance when wearing personal protective equipment including N95/FFP2 masks (Yánez Benítez et al., 2020). However, the performance was measured solely based on subjective reports from the surgeons involved.

The first studies using actual performance data show that in short cognitive performance tests, mask wearing had at most a very small effect on performance (Spang and Pieper, 2021). Similarly, heart rate variability and blood oxygen saturation showed only very slight (non-significant) changes.

However, many cognitive performance assessments in the context of personnel selection take several hours to complete. So far, the influence of mask wearing over a long period of time on performance has not been investigated. The effects of mask wearing on performance could become significant in longer testing sessions, for example by reduced oxygen uptake, increased discomfort or obstruction in the execution of the test.

During the pandemic, face masks were a central component not only of health and safety measures but also of extensive operational hygiene concepts for unavoidable encounters in a work-related setting. They were used to reduce the spread of the Covid-19 virus in face-to-face situations. However, the fact that they cover the wearer’s mouth and nose raises concerns that interpersonal communication is impaired. Mask wearing could influence the observability of emotions and competencies in interactive selection processes. As these exercises rely on interpersonal interaction, masks could interfere with the candidates’ observed performance.

A growing number of studies investigated the impact of different types of face masks on various aspects of communication. Bonnell (2020) identified six ways in which masks can obstruct communication, which can be broken down to two factors: verbal communication and emotion detection.

In general, face masks reduce the volume of vocal speech, especially in the higher frequency range (Corey et al., 2020; Magee et al., 2021). This can reduce the intelligibility of verbal communication. For example, the average intelligibility threshold at which spoken words are understood is raised by 12.4 dB with N95 masks (Bandaru et al., 2020). Although it was not found that overall speech quality is reduced or that intelligibility is substantially compromised under controlled laboratory conditions (Magee et al., 2021), it was shown that intelligibility is impaired under non-optimal conditions with surgical masks, specifically when mixed with competing speech signals (Smiljanic et al., 2021). In addition to some acoustic features of the spoken word, face masks also hide important visual cues (e.g., lip reading) that normally help us understand verbal messages (Atcherson et al., 2021). Infants have great difficulties recognizing spoken words if the speaker wears a mask but not if the speaker wears a transparent one (Singh et al., 2021). This could further complicate the perception of purely verbal communication. People usually adapt to novel circumstances and tend to speak louder and more clearly with masks to compensate for the difficulties caused by the mask (Magee et al., 2021; Smiljanic et al., 2021). Over time, however, this could lead to verbal communication requiring more effort and being more exhausting (Ribeiro et al., 2020).

Interactive exercises in an AC may be more challenging with mask-related acoustic problems (Corey et al., 2020; Magee et al., 2021; Smiljanic et al., 2021). Communication between participants may be more protracted, while misunderstandings may occur more frequently. Assessors might likewise miss more details, causing observation accuracy to deteriorate and, in the worst case, misconstrue this as poor communication skills on the part of the candidate. Candidates may try to compensate for increased difficulty communicating by speaking louder and more clearly (Smiljanic et al., 2021), thus putting in more effort, which might reduce mental capacities and performance in other areas (Ribeiro et al., 2020).

In addition to the verbal aspect of communication, wearing a face mask also precludes parts of the facial expressions that are essential for emotion recognition and nonverbal communication. When individuals wear a mask, emotion detection by an observer is slower (Williams et al., 2021) and the accuracy is reduced (Carbon, 2020; Grundmann et al., 2021). This is especially pronounced when detecting a positive or negative emotion, but not when detecting a neutral facial expression as these are often decoded with sufficient accuracy just by looking at the eyes (Marini et al., 2021).

Interestingly, masked faces are generally perceived as more trustworthy than unmasked faces (Marini et al., 2021). This means that happy faces with masks are rated as being just as trustworthy as unmasked happy faces. However, individuals with negative emotional expressions are perceived as less untrustworthy when wearing a face mask than when not wearing one (Grundmann et al., 2021; Marini et al., 2021). It is possible that positive emotion is decoded more strongly via the eye areas, while negative emotion is conveyed more through the mouth area and the rest of the face (Carbon, 2020). Similarly, it is possible that the ambiguity caused by missing information normally conveyed via facial expressions leaves room for a positivity bias (Grundmann et al., 2021).

The difficulties in reading faces and detecting emotions affect candidates and assessors alike. Candidates’ performance in AC exercises may be impaired by inaccurate emotion detection and social judgement (Grundmann et al., 2021) of other candidates in group exercises or professional role players in standardized social situations. Inappropriate behavior resulting from misperception as a result of mask wearing may be interpreted by assessors as inadequate social skills. In addition, the assessors themselves have problems accurately assessing the emotions and behaviors of the candidates, since part of the face is covered by the mask.

Health and safety measures – specifically mask wearing – in a long-duration high-stake situation may have a potential impact both on candidates’ performance as well as observers’ assessment accuracy and thus on fairness (Urbina, 2014) of the selection procedure. Therefore, this study investigates the question of whether candidates who complete their selection process on-site under the conditions of health and safety measures with face masks (2021) and without a face mask (2020) have comparable chances of passing the different stages of a selection process and are able to achieve the same results as candidates tested and assessed under the non-pandemic condition (2019). Candidates’ results in standardized cognitive tests as well as AC exercises will be compared across these three conditions.

Among all the health and safety measures, the mask mandate might have an exceptional influence on behavioral observation. Therefore, the face mask’s potential impact on the subjectively perceived quality of behavioral observation by the assessors is evaluated. It will be investigated whether the assessors thought they were able to observe behavior equally well with the health and safety measures as without.

The study was realized in cooperation with the German Federal Police’s aviation school.

In separate analysis steps it will be examined, whether, respectively, to what extent mask wearing and the other health and safety measures have an influence on.

1. The pass rate in the three selection stages (after cognitive testing, after AC, after the concluding interview).
2. Candidates’ performance in the different cognitive tests.
3. Candidate’s performance in the AC tasks role play and dyadic cooperation test (DCT).

Further it will be analyzed to what extend mask wearing has an influence on.

4. Assessors’ subjective observability of the areas of competence (AOC) in role play, DCT and interview.

## 2. Materials and methods

### 2.1. Procedure

Two sources of information provided the data basis for the analyses.

- Performance data of all German Federal Police’s aviation school helicopter applicants were compared across the years 2019 (pre-pandemic), 2020 (health and safety measures without face mask), and 2021 (health and safety measures with face mask), taking into account cognitive performance testing and AC as well as the final results (after interview).
- A questionnaire was developed in which 14 AC assessors and 5 specialized DCT assessors were asked to rate the extent to which wearing a face mask (campaign 21) might have affected observability during behavioral observation. Assessors were asked to indicate the observability of each area of competence they had to observe during the different exercises.

The team of AC assessors consisted of 10 aviation psychologists and 4 helicopter flight instructors from the German Federal Police. All assessors underwent observer training and participate regularly in selection assessments. The team of DCT assessors consisted of 5 qualified psychological technical assistants extensively trained in behavioral observation for the DCT.

### 2.2. Personnel selection at DLR

The German Aerospace Center (DLR) carries out selections for operational aviation personnel like pilots and air traffic controllers as well as astronauts on an international scale.

Different selection procedure components are used for different target professions. Since the present study was carried out in cooperation with the German Federal Police’s aviation school, the relevant procedures for helicopter pilots will be briefly described here.

The psychodiagnostic selection is structured into three stages:

Stage 1: Computerized aptitude testing (CAT) of cognitive and psychomotor abilities as well as basic knowledge. This first stage of pilot selection is spread across one whole day.
Stage 2: The Assessment Center (AC) consists of a role-play and a dyadic cooperation test (DCT) which is a work sample team test.
Stage 3: The interview makes up the final component of the selection procedure. It is semi-structured, using a set of guidelines and lasts 60 to 90 min.

Candidates who did not pass Stage 1 (CAT) were not invited to Stage 2 (AC). Candidates who failed to pass in Stage 2 were not eligible for the final interview (Stage 3).

Criteria for evaluating candidate’s aptitudes and for pass/not pass-decisions did not change over the years in all three stages. Selection was at no point quota-driven, as is reflected in the fact, that each year more candidates were assessed positive than needed.

#### 2.2.1. Cognitive tests

In the first stage of the selection procedure candidates performed computer-based tests in a group setting. All tests were conducted on the same day in a fixed order. The test protocol contained tests for cognitive mental ability, knowledge skills, psychomotor and multitask ability. The entire CAT test protocol lasts about 8 h.

*Spatial ability* included one test for spatial visualization in which candidates had to decide which of five possible dice matches a given unfolded dice (Zierke, 2014) and one for spatial orientation where candidates had to count the number of either left or right turns of a progressing path.

*Visual perception* was assessed with one test in which candidates had to quickly read the numbers on nine dials and subsequently reproduce the correct values. Only some of the nine dials were target dials, determined by either color or shape of the dials (Zierke, 2014).

In the test for *concentration*, the task was to compare a series of successive triangles in a short time with regard to various characteristics (e.g., color, orientation) and to press a different button depending on the response. As this test was revised after campaign 19, only comparisons between campaigns 20 and 21 were available.

One test assessed *auditory memory* with a running memory span test (Zierke, 2014). Candidates had to memorize an acoustically presented sequence of digits and enter them in reverse order.

In the test for *pattern recognition*, candidates had to choose which of five presented geometric shape can be found in a complex pattern.

For the knowledge skills domain, four tests assessed *English language*, *mechanical comprehension*, *math* (consisting of mental calculation and more complex math) (Zierke, 2014) requiring candidates to answer items from the different knowledge domains.

Lastly, for *hand-eye-coordination, hand-foot-eye-coordination* and *multitask ability*, two monitoring and instrument tests were carried out. The first test resembled a very basic flight simulator and requires candidates to control different parameters (heading, altitude, speed) with either the joystick or a button press. The performance in the joystick tracking of heading and altitude determined the performance for hand-eye-coordination.

In a second task, candidates had to use a joystick, throttle, and foot pedals to coordinate control of three parameters simultaneously. The performance in this test determined hand-foot-eye-coordination ability. For the multitask ability, the previously mentioned tasks were supplemented by additional attention tasks (e.g., monitoring sequences of numbers). To evaluate multitask ability, the results of both tests were averaged.

For more information about reliability and structural interrelations of the aforementioned tests, see Hermes and Stelling (2016) and Hermes et al. (2019).

#### 2.2.2. Assessment center

The AC comprised a role play and the DCT. The role play was a one-on-one interaction exercise conducted with one candidate and one trained role player. The role plays were conflict-oriented and last 10 min. A dilemma situation required the candidate to deal with a disgruntled role player.

The DCT demanded crucial characteristics of cockpit teamwork (Stelling, 1999). Candidates had to cooperatively manage a complex traffic management system. The entire procedure including instruction and practice takes 90 min. For more detailed description of the tasks see also Zinn et al. (2020). Focused competencies during the Assessment Center tasks were stress resistance, rule fidelity, decision making, assertiveness and team orientation.

In role play and DCT the observation processes were highly structured. In role play, areas of competence were exactly defined, with distinct examples of behavior for specific situations. DCT observation was even more structured: For each sequence (between 1 and 5 min in length), relevant behavioral units are counted in an observation plan.

#### 2.2.3. Interview

The interview was a standardized and semi-structured conversation with the candidate. It was hypothesis-driven and referred partially to the performance in the AC areas of competence in the sense that weaker performed areas were addressed more thoroughly. Furthermore, candidate’s achievement motivation, job motivation, and communicative skills were assessed. Candidates´ biography and the self-reported personal strengths and weaknesses were also reviewed. The interview lasted 60 to 90 min.

The subsequent and final assessors´ discussion then considered all selection stages and led into an overall risk assessment for each of the AOC. Therefore, candidate’s interview performance was not rated separately but was reflected in the overall pass rates.

### 2.3. Health and safety measures

In April 2020 conditions were established and adapted during the pandemic to ensure the safety of everybody involved in the selection process. At the same time the selection process had to be comparable with pre-pandemic years.

In coordination with the DLR crisis management team and subject to the Ordinance on the Containment of the Spread of the Coronavirus SARS-CoV-2 in the Free and Hanseatic City of Hamburg from April 2nd, 2020, a hygiene plan was drawn up for the first time in April 2020. The hygiene concept was implemented in this first version until the end of the selection campaign in October 2020 (Campaign 20). The hygiene concept was later revised in February 2021, adapting to new and in many aspects stricter official regulations. For both years, hygiene concepts included (but not limited to) the following health and safety measures:

1. Those involved in the tests were required to affirm that they had not neither visited a high-risk area nor had had contact with infected persons during the previous 14 days.
2. Candidates were required to wear face masks in corridors, waiting rooms, and restrooms.
3. Candidates had to maintain a distance of 1.50 m at all times. This led to a lower test capacity in test rooms, as well as limited use of waiting rooms and elevators.
4. Candidates were asked to wash or disinfect their hands before starting the test.
5. Lockers and water dispensers were unavailable. Candidates were informed in advance of the examination that they were to bring food and drinks with them for the day of the examination.

Compared to the 2020 version, the hygiene concept for 2021 was altered to reflect changes to the health and safety regulations:

1. Candidates now had to wear a face mask (surgical or FFP2 mask) during all parts of selection.
2. FFP2 masks were mandatory in corridors and during breaks.
3. The number of participants permitted during each examination was reduced by approximately 15%.

### 2.4. Assessor questionnaire

All assessors were asked in 2021 to retrospectively assess their experience with the health and safety measures. Reference was only made to the 2021 campaign, as it was only mandatory in this year to wear a mask during the exercises.

Assessors were asked to rate each area of AC competence regarding the observability for candidates wearing face masks compared to pre-pandemic times, on a scale from −4 to 4. On this scale, a rating of 0 meant equivalent observability, while values below 0 meant higher observability without mask and above 0 meant higher observability with a mask.

Assessors were additionally asked to indicate on a scale from 0 to 5 to what extent the masks interfered with their acoustic perception and if they found the mask distracting.

### 2.5. Participants

The number of participants per stage and year are presented in Table 1 alongside mean age and standard deviation.

**Table 1:** Number, mean age, and standard deviation of age of candidates per selection stage and year.

	Campaign 19			Campaign 20			Campaign 21		
	Number	M_Age_	SD_Age_	Number	M_Age_	SD_Age_	Number	M_Age_	SD_Age_
CAT	186	25.65	5.43	200	25.20	5.01	258	25.71	4.28
AC	80	24.19	4.25	79	25.10	4.86	148	24.37	4.31
Interview	49	24.53	4.34	56	24.59	4.69	99	24.29	4.25

Age of participants did not significantly differ between the three campaigns in CAT (*F*(2,641) = 0.679; *p* = 0.507), AC (*F*(2,304) = 0.983; *p* = 0.376), nor Interview (*F*(2,201) = 0.098; *p* = 0.907).

### 2.6. Analyses

For comparing pass rates in the different campaigns, we computed χ^2^ tests for the selection steps after CAT, AC and Interview, respectively.

In order to determine the effect of health and safety measures – and mask wearing in particular – on performance in various different cognitive, psychomotor and skills tests (CAT), we computed a single-factor MANOVA with campaign as factor and all CAT tests as variable complex. Upon a significant MANOVA, we were to compute additional single-factor ANOVAs for each CAT test.

For AC exercises (role play and DCT), we computed single-factor MANOVAs combining the areas of competence: stress resistance, decision making, cooperation, assertiveness (only role play) and rule fidelity (only DCT). In case of significance, single-factor ANOVAs were computed as a *post hoc* measure.

Finally, we analyzed the subjective observability of each of the areas of competence, as indicated by the assessors and DCT observers. Ratings from 1 to 9 were tested in a one-sample *t*-test against the score 5 (i.e., no subjective difference between with and without a face mask) to determine subjective differences in any of the given items.

## 3. Results

### 3.1. Pass rates

We computed separate χ^2^ tests for Stage 1 (CAT tests) on the one hand and for Stages 2 and 3 (AC and interview) on the other because some of the candidates participating in Stages 2 and 3 in 2019 through 2021 might have passed their Stage 1 assessment in the years prior to 2019.

#### 3.1.1. Stage 1: CAT

The number of candidates with a positive and with a negative result in stage 1 (CAT) by year are presented in Table 2. No significant difference with regard to the final pass rate could be found (χ^2^(2) = 3.210; *p* = 0.201). Positive and negative results were relatively equally distributed among the campaigns. Hygienic rules without masks (campaign 20) and with masks (campaign 21) had no relevant influence on passing rates of Stage 1 testing.

**Table 2:** Number of participants with a positive or negative result in stage 1 testing by year.

	Number of candidates		
	Positive	Negative	Total
Campaign 19	85	101	186
Campaign 20	103	97	200
Campaign 21	140	118	258

#### 3.1.2. Stage 2: assessment center and final decision

Table 3 presents the number of candidates who received a negative result after the AC, the interview or who received a positive final result. No significant differences with regard to the AC pass rate and the final pass rate could be found (χ^2^(4) = 0.701). Positive and negative results were relatively equally distributed among the campaigns for the selection stage after AC and for the selection stage after the interview (final decision). Health and safety measures without masks (campaign 20) and with masks (campaign 21) had no significant influence on either result.

**Table 3:** Number of participants with a negative result in AC, interview or a positive final result by year.

	Number of candidates			
	Positive	Negative AC	Negative interview	Total
Campaign 19	34	31	15	80
Campaign 20	40	23	16	79
Campaign 21	74	49	25	148

### 3.2. Cognitive aptitude testing – mean comparison

Means and standard deviations for each test by campaign year is presented in Table 4. Scores were transformed to *T*-values with 50 being the average and 10 the standard deviation.

**Table 4:** Means and standard deviations as standardized *T*-values for all areas of performance in CAT.

	Campaign 19	Campaign 20	Campaign 21
	*M* (SD)	*M* (SD)	*M* (SD)
Spatial ability	49.85 (8.44)	50.98 (8.67)	51.37 (8.69)
Visual perception	50.40 (9.97)	51.90 (10.18)	51.01 (10.58)
Concentration	–	47.70 (7.97)	47.48 (8.59)
Auditory memory	48.39 (9.28)	49.80 (9.39)	50.31 (10.93)
Pattern recognition	50.11 (9.31)	49.35 (9.37)	50.89 (9.66)
English language	52.55 (9.01)	53.68 (8.89)	54.55 (9.45)
Mechanical comprehension	48.53 (9.24)	48.49 (8.18)	49.22 (9.14)
Math	48.35 (8.24)	48.39 (7.90)	48.58 (8.51)
Hand-eye-coordination	50.40 (9.88)	48.75 (10.44)	50.04 (10.21)
Hand-foot-eye-coordination	49.33 (10.96)	50.00 (10.81)	50.64 (11.40)
Multitask ability	50.36 (9.31)	50.33 (9.91)	51.67 (9.82)

A single-factor MANOVA was computed with the campaign as the independent variable and all areas of performance except Concentration as the variable complex. Results of the concentration test registered in campaign 2019 could not be included in the comparative analysis; due to the test revision between campaigns 2019 and 2020 the raw data dimensions changed to a different measurement standard. For the more important comparison of campaign 2020 (health and safety measures without mask wearing) with campaign 2021 (health and safety measures with mask wearing) a *t*-test for independent samples was calculated.

The MANOVA showed no statistically significant difference between the campaigns on the combined Areas of Competence as dependent variables, *F*(20, 264) = 1,241, *p* < 0.211, partial η^2^ = 0.019, Wilk’s Λ = 0.962. Regarding Concentration there was also no significant difference between campaign 2020 and campaign 2021, *t*(456) = 0.280, *p* < 0.780.

### 3.3. Assessment center

For both AC tasks (role play and DCT) the influence of the health and safety measures on candidates’ performance in each area of competence was analyzed.

#### 3.3.1. Role play

In role play, four areas were observed: stress resistance, decision making, assertiveness and teamwork skills. Rating scores range from 1 to 6. Means and standard deviations for each area of competence by campaign are presented in Table 5. Levene tests were performed for all four dimensions to examine the difference in variances of the values for the respective campaigns. Neither Stress resistance (*F*(2,304) = 0.612; *p* = 0.543), nor Decision Making (*F(2,304)* = 1.627; *p* = 0.198), Assertiveness (*F*(2,304) = 0.397; *p* = 0.673), or Teamwork skills (*F*(2,304) = 0.866; *p* = 0.422) showed significant differences in the variance of the ratings.

**Table 5:** Means and standard deviations as standardized *T*-values for areas of competence in the role play.

	Stress resistance	Decision making	Assertiveness	Teamwork skills
Campaign 19	48.78 (9.33)	50.49 (10.63)	50.58 (10.02)	49.50 (10.58)
Campaign 20	51.65 (10.23)	51.44 (9.03)	50.97 (9.48)	51.25 (9.81)
Campaign 21	49.75 (10.08)	49.00 (10.09)	49.15 (10.30)	49.67 (9.78)

A single-factor MANOVA was computed with the campaign as the independent variable and areas of competence from the role plays as the variable complex. The MANOVA showed no statistically significant difference between the campaigns on the combined Areas of Competence as dependent variables (*F*(8, 602) = 1,345, *p* = 0.218, partial η^2^ = 0.018, Wilk’s Λ = 0.965).

#### 3.3.2. Dyadic cooperation test

In the DCT, four areas were observed: stress resistance, decision making, rule fidelity and teamwork skills. The means and standard deviations of each area of competence by campaign is shown in Table 6. Scores for each area range from 1 to 6. Levene tests were performed for all four dimensions to examine the difference in variances of the values for the respective campaigns. Neither Stress resistance (*F*(2,304) = 0.081; *p* = 0.922), nor Rule Fidelity (*F*(2,304) = 0.851; *p* = 0.428) or Decision Making (*F*(2,304) = 0.815; *p* = 0.444) showed significant differences in the variance of the ratings. Only the variances for Teamwork skills (*F*(2,304) = 11.493; *p* < 0.001) were significantly different, i.e., higher in the campaigns with health and safety measures.

**Table 6:** Means and standard deviations as standardized *T*-values for areas of competence in the DCT.

	Stress resistance	Rule fidelity	Decision making	Teamwork skills
Campaign 19	50.71 (9.78)	49.98 (9.55)	50.37 (9.43)	47.10 (7.63)
Campaign 20	49.05 (9.96)	49.75 (9.40)	48.63 (9.32)	49.92 (9.99)
Campaign 21	50.17 (10.23)	50.15 (10.64)	50.44 (10.65)	51.72 (10.69)

A single-factor MANOVA was computed with the campaign as the independent variable and the DCT areas of competence as the variable complex. The MANOVA showed a statistically significant difference between the campaigns on the combined areas of competence as dependent variables (*F*(8, 602) = 2,097, *p* = 0.034, partial η^2^ = 0.027, Wilk’s Λ = 0.947).

Since we could observe a significant main effect, separate single-factor ANOVAs were computed for each area of competence: Neither stress resistance (*F*(2,304) = 0.570; *p* = 0.566), rule fidelity (*F*(2,304) = 0.040; *p* = 0.961) nor decision making (*F*(2,304) = 0.935; *p* = 0.394) yielded significant differences. However, the ANOVA for teamwork skills was significant (*F*(2,304) = 5.775; *p* = 0.003). Post-hoc *t*-tests with TukeyHSD α-correction show that there is a significant difference (*p* = 0.002) in the rating of teamwork skills between campaign 19 and campaign 21. There were no significant differences between campaign 19 and campaign 20 (*p* = 0.167) as well as between campaign 20 and campaign 21 (*p* = 0.387).

### 3.4. Assessors questionnaire – subjective observability

In order to find out whether the assessors perceive an influence of candidates’ mask wearing on the observability of the areas of competence in role play and DCT, they were asked for each area of competence, on a scale from −4 to 4, if the areas of competence were *much less assessable with mask (−4), about equally assessable (0)* or *much more assessable with mask (4).*

#### 3.4.1. Role play

**Figure 1:**
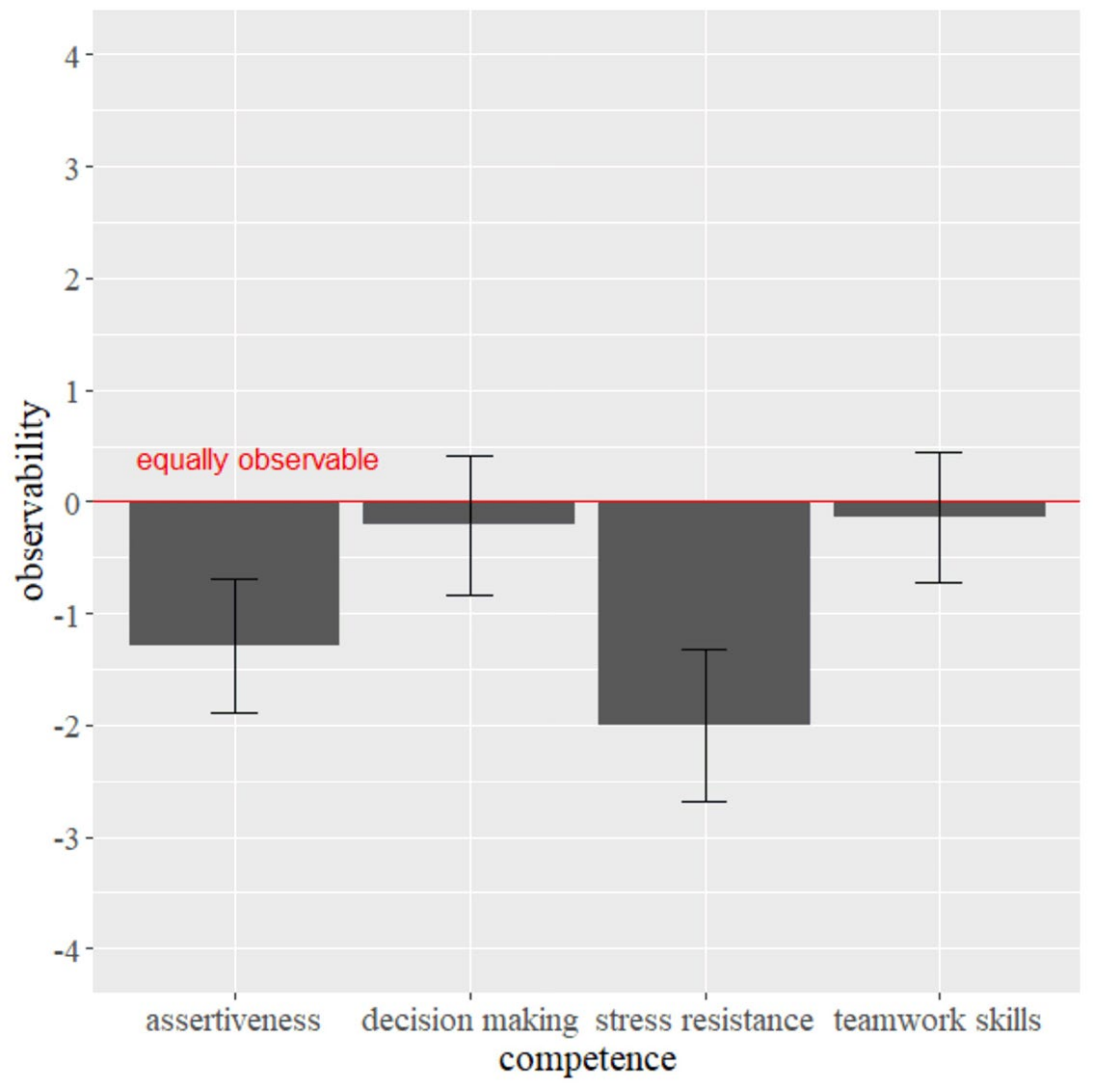
Subjective observability of AC areas of competence. Error bars indicate the 95% confidence interval.

Assessors were asked to rate all four areas of competence in the role play (stress resistance, decision making, assertiveness and teamwork skills) for the observability with and without candidates’ mask wearing. Figure 1 shows the means and standard errors of each area of competence.

Separate one-sample *t*-tests were used to determine whether the ratings of the 14 assessors were different from the value of 0 (“about equally assessable”).

Stress resistance (*t*(13) = −5.75; *p* < 0.001) and assertiveness (*t*(13) = −4.23; *p* = 0.001) were rated as significantly less observable when candidates are wearing masks while decision making (*t*(13) = −0.675; *p* = 0.512) and teamwork skills (*t*(13) = −0.486; *p* = 0.635) were not rated as significantly less observable with mask wearing than without mask wearing.

#### 3.4.2. Dyadic cooperation test

**Figure 2:**
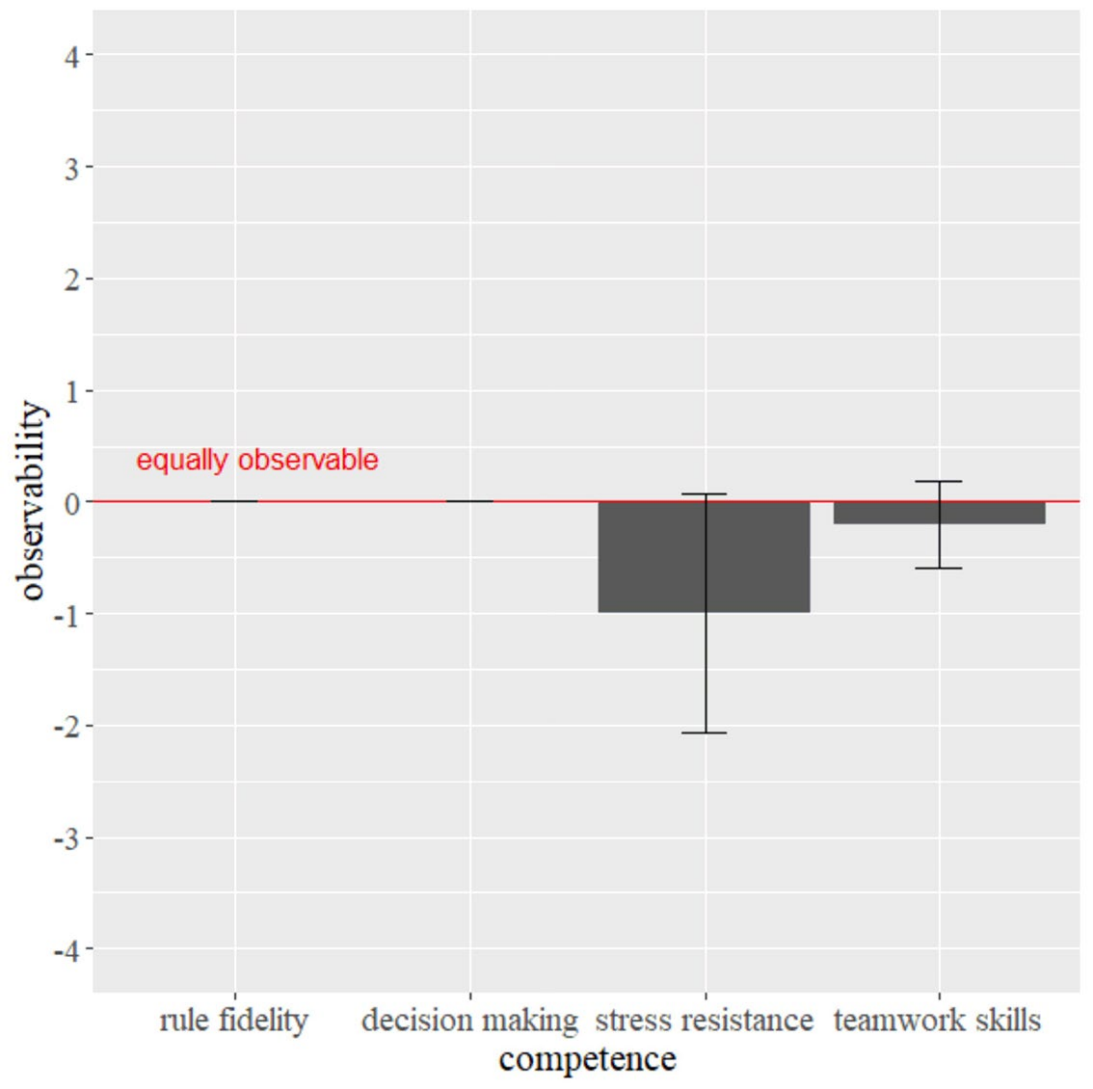
Subjective observability of DCT areas of competence. Error bars indicate the 95% confidence interval.

DCT assessors were asked to rate all four areas of competence in the DCT (stress resistance, rule fidelity, decision making and teamwork skills) for the observability with mask wearing. Figure 2 shows the means and standard errors of each area of performance.

All 5 DCT assessors rated rule fidelity and decision making as equally observable (value of 0). As there was no difference reported we could not calculate the significance. Separate one-sample *t*-tests were used to determine whether the ratings of the 5 DCT assessors were different from the value of 0 (“about equally assessable”) for stress resistance and teamwork skills.

Neither stress resistance (*t*(4) = −1.826; *p* = 0.142), nor teamwork skills (*t*(4) = −1.000; *p* = 0.374) were rated as significantly less observable with mask wearing in comparison to when candidates wear masks.

#### 3.4.3. Interview

In the final semi-structured interview, all areas of competence from the AC were assessed in conclusion. Three additional areas were assessed: communication, achievement motivation and job motivation.

**Figure 3:**
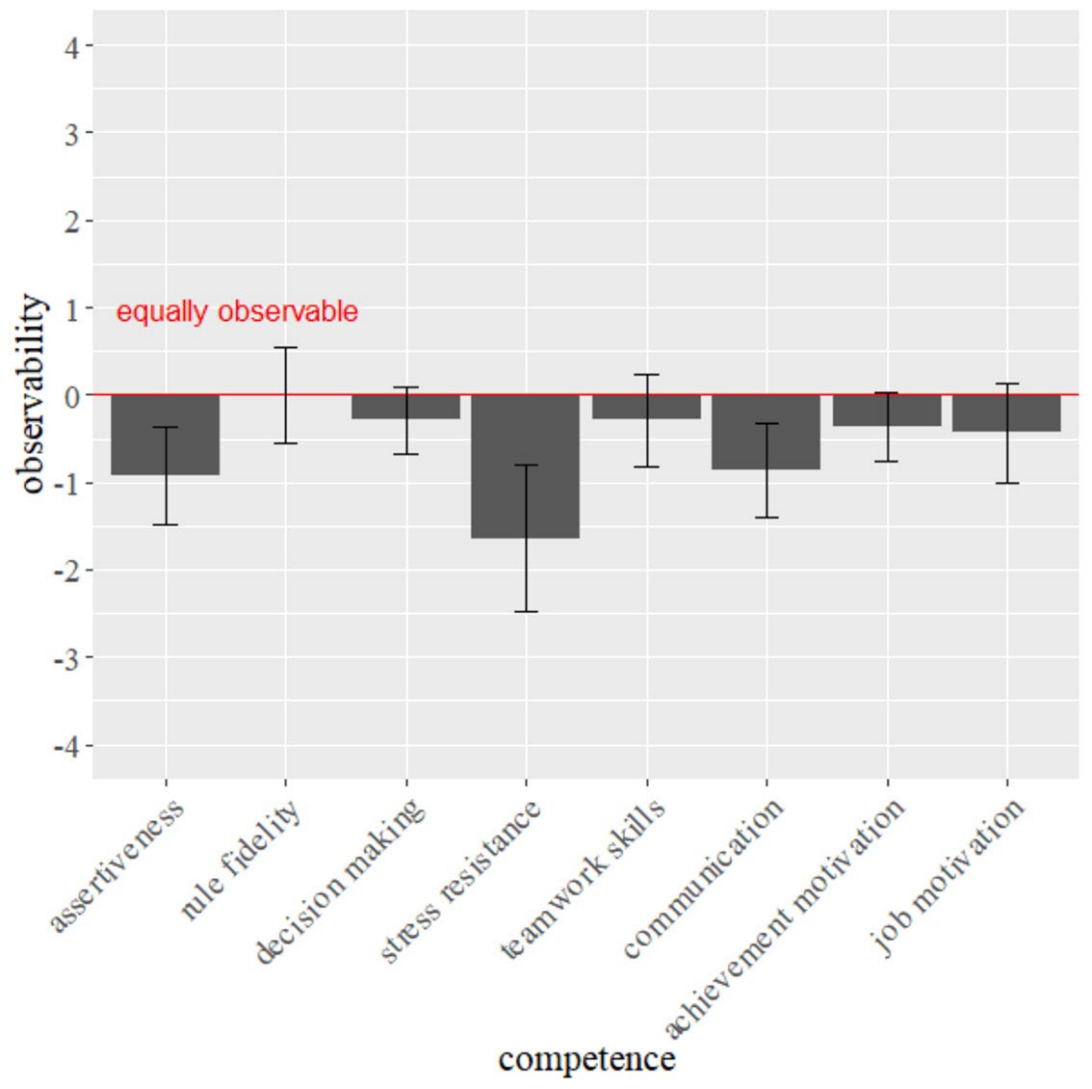
Subjective observability of interview areas of competence. Error bars indicate the 95% confidence interval.

The assessors were asked to rate all seven areas of competence in the interview for the observability with mask wearing. Figure 3 shows the means and standard errors of each area of performance.

Separate one-sample *t*-tests were used to determine whether the ratings of the 14 assessors were different from the value of 0 (“about equally assessable”). The results are shown in Table 7.

**Table 7:** Results of the one-sample *t*-tests for the observability of each area of competence observed in the interview.

	One-sample *t*-test		
	*t*	*df*	*p*
Stress resistance	−3.85	13	0.002**
Decision making	−1.47	13	1.65
Assertiveness	−3.24	13	0.006**
Teamwork skills	−1.08	13	0.302
Rule fidelity	0.00	13	1.000
Communication	−3.12	13	0.008**
Achievement motivation	−1.79	13	0.096
Job motivation	−1.47	13	1.65

**p < 0.01.

Assessors rated the three areas of competence stress resistance, assertiveness and communication as significantly less observable when candidates are wearing face masks. All other areas of competence were not rated significantly less observable with mask wearing.

#### 3.4.4. Distraction and acoustic effects

We asked all assessors how much they were distracted by the mask in their own performance and how much the mask interfered with their acoustic perception. Both ratings were tested against 0, meaning that no difference was perceived compared to pre-pandemic assessments and 5 meaning that performance, respectively, acoustic perception was strongly affected.

Assessors indicated that they were distracted in their work with a mean rating of 1.37 (SD = 1.21), which is a significant difference to pre-pandemic assessments (*t*(18) = 4.923; *p* < 0.001).

Regarding acoustic intelligibility, assessors indicated that masked candidates were more difficult to understand (M = 1.68, SD = 0.95), which was significantly different from pre-pandemic assessments (*t*(18) = 7.761; *p* < 0.001) and did not differ significantly between AC and DCT assessors (*t*(17) = 0.774; *p* = 0.450).

## 4. Discussion

Our findings suggest that overall pass rates in different stages of the selection procedure were unaffected by health and safety measures and mask wearing.

The results from the cognitive aptitude tests (CAT) also show that even objective performance data is unaffected by either health and safety measures with and without mask wearing. Not only were pass rates stable at this stage, but results in individual performance domains were comparable to pre-pandemic selection. CAT participants work on the computer-based tests for more than 8 h, albeit with breaks. Because participants in the 2021 campaign wore a mask for the entire duration of the test, our results on performance over a long period of time confirm preliminary findings on the equivalence between wearing a mask and not wearing a mask on short performance tests (Spang and Pieper, 2021). However, symptoms of physical discomfort (e.g., headache) were often reported when wearing masks. It is possible that these physical symptoms only affect the simplest performance parameters (e.g., simple reaction time) and have no significant influence on somewhat more complex tasks (Mittelstädt et al., 2019). Since only minor physiological changes (e.g., blood oxygen saturation) can be detected after mask wearing (Spang and Pieper, 2021), it is unlikely that masks impair performance overall, even if they are worn for a long period of time.

AC and final pass rates were also not significantly affected by wearing a face mask and the other health and safety measures. Our data further show that the ratings for individual competences assessed in the role play were comparable for pandemic and pre-pandemic selection.

Only in the dyadic cooperation test was there a significant difference in the sense that teamwork skills were rated better with mask wearing (2021) than before the pandemic (2019). The small increases between campaign 2019 and 2020 as well as between 2020 and 2021 both remained insignificant. Certainly, candidates may have simply got better at this area of competence in this specific dyadic task (and not in role play) over the years. However, it seems more likely that the masks, by limiting opportunity for exchanging facial expressions, led candidates to engage in more verbal exchanges and hence to offer more assistance (Yi et al., 2021). Likewise, the DCT assessors might have simply perceived the performance of the masked candidates more favorably (Marini et al., 2021), but this is unlikely given the structured procedure with fixed mostly verbal behavioral markers. In addition, DCT assessors rated teamwork skills as being about equally observable with and without a mask.

Overall, the DCT assessors saw considerably fewer impairments to subjective observability than did the role play and Interview assessors. One likely reason for this could be the even greater focus on verbal behavioral markers in the DCT. In addition, in this exercise the candidates are observed from behind from an approximately 20-degree angle and facial expression plays a subordinate role for the evaluation.

In the role play and Interview, competences were specifically considered to be less observable where either acoustic barriers have led to poorer comprehension (communication) or facial expressions are used to detect emotion (assertiveness and stress resistance). This is supported by the findings that (especially the AC) assessors felt their performance was distracted and rated acoustic intelligibility as impaired. These results are consistent with the assessment of surgeons who perceive their performance to be impaired in the areas of verbal communication and decision making when wearing protective gear including a face mask (Yánez Benítez et al., 2020).

In communication, the limitations of intelligibility due to mask use (Bandaru et al., 2020; Corey et al., 2020) will likely have meant that more effort was put into communicating, e.g., by repeating sentences or speaking louder (Magee et al., 2021). This increased effort, along with a lack of facial visual cues (Atcherson et al., 2021) and attenuated paraverbal signals, might have led assessors to rate communication ability as less observable.

Because people often use facial features and facial expressions to determine emotions, the observability of assertiveness and stress resistance is limited by facial masks. Assertive candidates may show more confident facial expressions with more relaxed muscle movements (Kolotkin et al., 1984). In contrast, stressed candidates show less relaxed facial expressions, including pursed lips, trembling or blushing (Lerner et al., 2007; Egawa et al., 2018).

Crucially, however, reduced reported observability did not result in significantly altered pass rates or ratings. A possibility is that, with greater uncertainty due to reduced observability, assessors retreat to mean ratings and reduce the variance of their assessments. However, in this structured role play setting the comparable level of standard deviation in the assessors’ ratings at least suggests that they did not rate with less variation and therefore rated with equal certainty.

Similar pass rates and ratings are not yet proof that candidates performed equivalently under the health and safety measures. Assessors may, for instance, have compensated for lower performance with more favorable ratings or given candidates the benefit of the doubt. However, assuming that the quality of candidates has remained constant across the years, equivalently high pass rates and ratings, as well as their variances, are a strong indication that on-site testing, even under mask use, entails the same precision and fairness as in non-pandemic selection. Provided that the selection process is standardized, the selection tools are reliable, and the assessors are well trained, companies should not let mandatory face mask regulations stop them from continuing onsite selection.

Although health and safety measures had a major impact on social interaction globally in almost all contexts, the findings of the current study show no noteworthy effects on our standardized personnel selection. Therefore, we suggest that practitioners should ensure that their selection instruments are standardized to the possible maximum in order to prevent interference with mask wearing. Then the subjective concerns about face masks in personnel selection can be neglected.

### 4.1. Limitations

Pandemics cannot be planned for. This study describes the health and safety measures that were devised out of health necessity and in the context of the state of knowledge at the time. However, applied health and safety measures were not randomized within the years. Thus, differences in the candidate pool of the campaigns could have influenced the results. Although we see no evidence for it, candidates from certain years may nevertheless have been more skillful or more motivated, for example.

Also, as mentioned above, in the AC we only compared the assessors’ ratings without knowing exactly whether the actual performance of the candidates differed. Behind this is again the assumption that the potential and general performance level of the candidates of the different campaigns did not differ.

### 4.2. Future research

It is quite possible that we will have to live with health and safety measures due to the Sars-Cov-2 for a little longer. Safety measures may become necessary periodically. A new pandemic will require the wearing of masks. The topic of mask-wearing will remain relevant when it comes to on-site personnel selection of the future.

Not only in personnel selection, but in psychological diagnostics in general, face masks could be an issue. Clinical diagnostics, in which reading of emotions is important for diagnosis, may be impaired by mask wearing. Despite the lack of differences in the outcome and the ratings given in our study, the assessors nevertheless indicated that they were less able to evaluate stress resistance and assertiveness. In a lesser structured diagnostic setting wearing of a face mask may as a result lead to an incorrect evaluation and hence in a clinical context to the wrong treatment approach. Future studies should focus on areas where psychological assessments also have to be performed on-site and health and safety measures could influence the quality of the diagnosis. Furthermore the degree of structure for observations should be varied and taken into account in future experimental designs (Hoermann and Goerke, 2014).

## Data availability statement

The datasets presented in this article are not readily available because since the data clearance of the participants only covers the handling by the authors and does not allow any disclosure to third parties, a publication is not available. Requests to access the datasets should be directed to FZ, frank.zinn@dlr.de.

## Ethics statement

Ethical review and approval was not required for the study on human participants in accordance with the local legislation and institutional requirements. The patients/participants provided their written informed consent to participate in this study.

## Author contributions

All authors listed have made a substantial, direct, and intellectual contribution to the work and approved it for publication.

## Conflict of interest

The authors declare that the research was conducted in the absence of any commercial or financial relationships that could be construed as a potential conflict of interest.

## Publisher’s note

All claims expressed in this article are solely those of the authors and do not necessarily represent those of their affiliated organizations, or those of the publisher, the editors and the reviewers. Any product that may be evaluated in this article, or claim that may be made by its manufacturer, is not guaranteed or endorsed by the publisher.
